# Airway epithelial cells initiate the allergen response through transglutaminase 2 by inducing IL-33 expression and a subsequent Th2 response

**DOI:** 10.1186/1465-9921-14-35

**Published:** 2013-03-13

**Authors:** Keunhee Oh, Myung Won Seo, Ga Young Lee, Ok-Jin Byoun, Hye-Ryun Kang, Sang-Heon Cho, Dong-Sup Lee

**Affiliations:** 1 Department of Biomedical Sciences, Laboratory of Immunology and Cancer Biology, Seoul, Korea; 2 Interdisciplinary Program of Cancer Biology, Cancer Research Institute, Seoul, Korea; 3 Transplantation Research Institute, Seoul, Korea; 4 Department of Internal Medicine, Seoul National University College of Medicine, Seoul, Korea; 5 Seoul National University College of Medicine, 103 Daehak-ro Jongno-gu, Seoul, Korea

**Keywords:** Epithelium, IL-33, Transglutaminase 2, Asthma, Animal models

## Abstract

**Background:**

Transglutaminase 2 (TG2) is a post-translational protein-modifying enzyme that catalyzes the transamidation reaction, producing crosslinked or polyaminated proteins. Increased TG2 expression and activity have been reported in various inflammatory conditions, such as rheumatoid arthritis, inflammation-associated pulmonary fibrosis, and autoimmune encephalitis. In particular, TG2 from epithelial cells is important during the initial inflammatory response in the lung. In this study, we evaluated the role of TG2 in the pathogenesis of allergic asthma, particularly whether TG2 affects initial activation signaling leading to Th2 differentiation against antigens.

**Methods:**

We induced allergic asthma by ovalbumin sensitization and intranasal challenge in wild-type (WT) BALB/c and TG2-deficient mice. Broncheoalveolar lavage fluid cells and intracellular cytokine production were analyzed by flow cytometry. Interleukin (IL)-33 and TG2 expression in lung epithelial cells was detected by confocal microscopy.

**Results:**

Airway responsiveness was attenuated in TG2-deficient mice compared to that in the WT control. In addition, recruitment of eosinophils and Th2 and Th17 differentiation decreased in TG2-deficient mice. Treatment with cysteamine, a transglutaminase inhibitor, also reduced airway hypersensitivity, inflammatory cell recruitment, and T helper cell differentiation. TG2-deficient mice showed reduced IL-33 expression following induction of allergic asthma compared to those in the WT control.

**Conclusions:**

We found that pulmonary epithelial cells damaged by allergens triggered TG2-mediated IL-33 expression leading to type 2 responses by recruiting both innate and adaptive arms of the immune system.

## Background

Allergic asthma is characterized by airway hyperreactivity, mucus hypersecretion, eosinophilic infiltration, and elevated serum IgE levels [1]. Although the early proposed role of abnormal airway smooth muscles has not been clearly defined, infiltration of inflammatory cells such as eosinophils, macrophages, and lymphocytes in the airways of patients with asthma and the efficacy of corticosteroids in the majority of patients indicate that asthma is a chronic airway inflammatory disease [2]. T cells play an important role during asthma pathogenesis, and T helper type 2 (Th2) cell differentiation is important in initiating and perpetuating events in asthma, particularly in experimental models [1,2]. The role of innate inflammatory cells, such as mast cells, basophils, and recently defined innate lymphoid cells, has been suggested to provide a local cytokine environment that induces Th2 differentiation [3]. In addition, epithelial cells at the mucosal surfaces have been accepted as integral components of innate and adaptive immunity [4,5]. The important role of pulmonary epithelial cells during lung infection has been documented [5-7], and the critical role of epithelial cells in inflammatory amplification following non-infectious damage has recently been reported by our group [8].

Transglutaminase 2 (TG2) is a post-translational protein-modifying enzyme that catalyzes the transamidation reaction, producing crosslinked or polyaminated proteins. TG2 is expressed ubiquitously in various cellular compartments and participates in many biological processes, including extracellular matrix formation, wound healing, apoptosis, and differentiation [9,10]. TG2 has also been implicated in many disease processes. Increased TG2 expression and activity have been reported in various inflammatory conditions, such as rheumatoid arthritis, gouty arthritis, and organ fibrosis [11-13]. Altered forms of proteins modified by TG2 enzymatic activity have been suggested in the pathogenesis of various diseases, such as celiac disease, cataracts, and Huntington’s disease [9,10,14-16]. Controversies exist regarding the pathological and protective roles of TG2 during inflammation, TG2 sustains inflammation through the release of inflammatory cytokines while minimizing inflammation by increasing the clearance of apoptotic cells [12,17]. Recent disease animal models using TG2-deficient mice have revealed the important role of TG2 during the pathogenesis of bacterial sepsis, inflammation-associated pulmonary fibrosis, and autoimmune encephalitis [8,18,19]. TG2 is important during the initial inflammatory response. Specifically, TG2 induces nuclear factor-κB-dependent interleukin (IL)-6 secretion from lung epithelial cells, leading to Th17 differentiation in the lung [8]. Epithelial cell-derived signaling mediators, such as IL-33, thymic stromal lymphopoietin (TSLP), and IL-25, initiate Th2 immune responses and each can direct the Th2 response either alone or through downstream mediators [4]. Among these, IL-33 has been implicated as the most upstream mediator of epithelial cytokines [4]. Since epithelial TG2 can initiate the inflammatory response of non-infectious tissue damage [8], we assumed the TG2 may also play an important role in initiating and perpetuating the epithelial inflammatory response leading to Th2 differentiation.

In this study, we investigated the role of TG2 in the pathogenesis of allergic asthma, particularly whether TG2 affects initial activation signaling by inducing IL-33 and downstream molecules leading to Th2 differentiation against antigens. Allergic asthma was induced by ovalbumin (OVA) sensitization and intranasal challenge. We found that airway hypersensitivity was attenuated in TG2-deficient mice compared to that in wild-type (WT) controls and recruitment of eosinophils and Th2 and Th17 differentiation was decreased in TG2-deficient mice. Treatment with cysteamine, a transglutaminase inhibitor, also reduced airway hypersensitivity, inflammatory cell recruitment, and T helper cell differentiation. TG2-deficient mice revealed decreased IL-33 expression following the induction of allergic asthma compared to that in the WT control. Thus, we provide evidence that TG2 in pulmonary epithelial cells initiates allergic responses by inducing the IL-33-Th2 signaling pathways.

## Materials and methods

### Mice

BALB/c mice were obtained from the Jackson Laboratory (Bar Harbor, ME). TG2^−/−^ mice were backcrossed to BALB/c mice for 12 generations (N12). Female, 8-12-week-old mice were used for experiments. All animal experiments were performed with the approval of the Institutional Animal Care and Use Committee at Seoul National University (authorization no. SNU05050203).

### Immunization

Mice were intraperitoneally administered phosphate buffered saline (PBS) containing OVA (grade V, Sigma-Aldrich, St Louis, MO) and aluminum hydroxide (alum) (Sigma-Aldrich) (20 μg OVA + 2 mg alum) two times at a 7-day interval. Intranasal OVA challenge (50 μg) was performed for 3 consecutive days starting on days 14 and 21 after the first immunization. Mice were injected intraperitoneally with cysteamine (40 mg/kg/day, Sigma-Aldrich) to inhibit TG2 activity.

### Airway responsiveness

Airway responsiveness was assessed as a change in airway function after challenge with aerosolized methacholine (Sigma-Aldrich) via the airways. Mice progressively inhaled 6.25–50 mg/ml methacholine for 5 min at 24 h after the last OVA intranasal challenge. Airway responsiveness was measured using the OCP3000 instrument (One Chamber Plethysmography for animals; Allmedicus, Anyang, Gyeonggi-do, Korea).

### Histopathology and immunofluorescence of lung tissue

Lung tissues were fixed in 4% paraformaldehyde, pro-cessed, and embedded in paraffin. Sections were stained with H&E for histopathological analysis. To investigate IL-33 expression in epithelial cells, lung tissue from airway hypersensitivity-induced mice were stained with anti-IL-33 (R&D Systems, Minneapolis, MN), anti-TG2 (Neomarker, Fremont, CA), and anti-pro-surfactant protein-C (pro-SP-C, Millipore, Billerica, MA). Alexa 488-conjugated donkey anti-goat IgG and Alexa 546-conjugated anti-rabbit IgG antibodies (Invitrogen, Carlsbad, CA) were used for visualization. Image acquisition and processing was performing using a confocal fluorescence microscope (Olympus, Center Valley, PA) and FV10-ASW 2.0 Viewer (Olympus).

### Analysis of broncheoalveolar lavage fluid (BALF)

Broncheoalveolar lavage was performed with five 1.0-mL aliquots of PBS through a tracheal cannula. Cytospin specimen was obtained and the cells were stained with Wright-Giemsa. BALF were analyzed for IL-13 and IL-17 and IL-4 and IFN-γ levels by sandwich ELISA. To evaluate cytokine production, cells were restimulated with 50 ng/ml PMA and 1 μg/ml ionomycin (Sigma-Aldrich) for 4 h. For intracellular staining, Golgi plug (BD-Pharmingen, San Diego, CA) was added during the final 2 h of stimulation. Cells were labeled with anti-CD4, anti-IL-4, anti-IL-13, anti-IL-17, and anti-IFN-γ antibodies (eBioscience, San Jose, CA). Intracellular cytokine levels were analyzed using a FACSCalibur (BD Biosciences, San Jose, CA) and FlowJo software (Tree Star, Ashland, OR).

### Statistical analysis

Statistical significance was analyzed using the Student’s *t*-test. A *p* value of < 0.05 was taken to indicate statistical significance.

## Results

### Airway inflammation was attenuated in TG2-deficient mice

**Figure 1:**
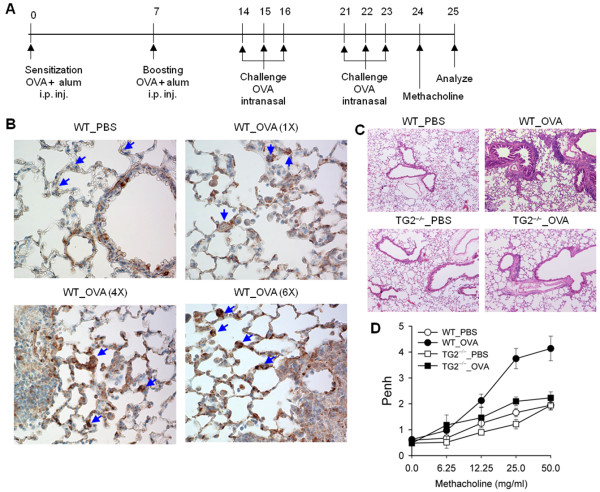
**Transglutaminase 2 (TG2) deficiency attenuates airway hypersensitivity.** (**A**) Wild-type (WT) and TG2 deficient (TG2^−/−^) mice were immunized with ovalbumin (OVA) plus aluminum hydroxide (alum) and intranasally challenged with OVA. (**B**) Immunohistochemistry of TG2 expression in the lungs from WT mice. Sections were prepared 24 h after intranasal OVA challenge (original magnification, ×400). (**C**) Representative photographs of lungs from WT and TG2^−/−^ mice after sensitization and challenge with PBS or OVA. Sections were stained with hematoxylin and eosin (original magnification, ×100). (**D**) Airway responsiveness was measured in sensitized and challenged mice. Pause was recorded for 3 min after aerosol methacholine treatment. The results for each group are expressed as means ± standard errors (n = 5).

WT BALB/c and TG2-deficient mice with a BALB/c background (N12) (TG2^−/−^) were intraperitoneally administered OVA and alum (20 μg OVA + 2 mg alum) two times at a 7-day interval, and an intranasal OVA challenge (50 μg OVA) for 3 consecutive days starting on days 14 and 21 to evaluate the role of TG2 during the pathogenesis of allergic asthma. Airway hyperresponsiveness (AHR) was assessed by methacholine challenge 1 day following the last intranasal OVA challenge, and the whole body method was used to measure enhanced pause. Bronchoalveolar lavage (BAL) fluid and lung tissue were sampled the next day (Figure 1A). TG2 expression from parenchymal lung tissue, especially from type II alveolar cells increased in OVA-immunized and -challenged mice compared to that in unmanipulated control mice (Figure 1B). The histopathological analysis of lung tissue following disease induction revealed that TG2^−/−^ mice had reduced airway inflammation with decreased inflammatory cell infiltration surrounding the airways as compared with WT mice (Figure 1C). Airway hyperresponsiveness also decreased in TG2 mice compared to that in WT mice (Figure 1D).

### Recruitment of eosinophils is reduced in TG2-deficient mice

**Figure 2:**
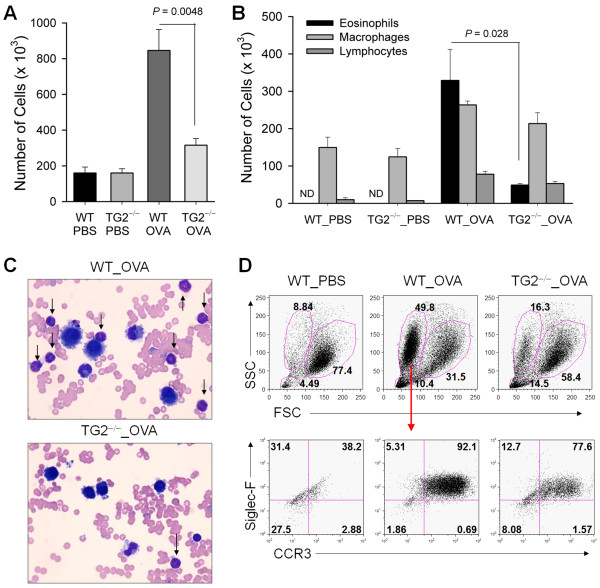
**Transglutaminase 2 (TG2) deficiency reduces eosinophil recruitment.** (**A**–**C**) Bronchoalveolar lavage (BAL) fluid from WT and TG2^−/−^ mice was obtained 48 h after the last OVA challenge. BAL cells were counted and analyzed by Wright-Giemsa staining. Total (**A**) and differential cell counts (**B**) were performed. (**C**) Representative photographs of BAL cells. Cells were stained with Wright–Giemsa (original magnification, ×1000). Arrows present eosinophils. (**D**) Inflammatory cells in BAL fluid from WT and TG2^−/−^ mice were labeled with anti-CCR3 and anti-Siglec-F antibodies and analyzed by flow cytometry. (**A**-**B**) Data are means ± standard deviation of three independent determinations with BAL cells from n = 5 mice/group.

OVA-sensitized and –challenged WT mice showed increased numbers of inflammatory cells in BAL fluid compared to those in the PBS-treated control mice. In addition, TG2^−/−^ mice showed decreased inflammatory cell infiltration compared to that in WT mice (Figure 2A). Cytospin analysis with Wright–Giemsa stain revealed that TG2^−/−^ mice showed a dramatic decrease in eosinophils, but not macrophages or lymphocytes, in BAL fluid. Thus, the reduction in infiltrating inflammatory cells in these mice reflected selective reduction of eosinophils (Figure 2B and 2C). We also confirmed a selective decrease of eosinophils in the TG2^−/−^ mice as compared with WT mice using flow cytometric analysis of BAL fluid (Figure 2D).

### Reduced Th2/Th17 differentiation in TG2-deficient mice

**Figure 3:**
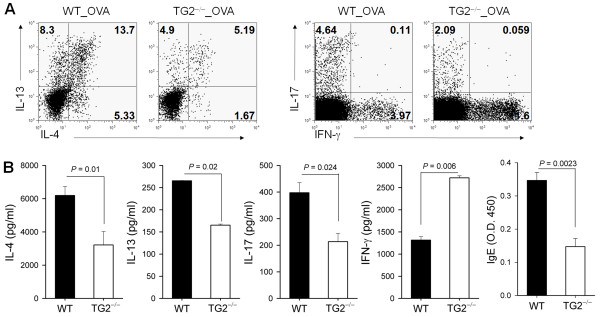
**TG2 deficiency reduces Th2 and Th17 differentiation.** (**A**) BAL cells were harvested 48 h after the last OVA challenge and restimulated with phorbol myristic acid (PMA) and ionomycin for 4 h. Intracellular cytokine levels were analyzed by flow cytometry. (**B**) Levels of interleukin (IL)-4, IL-13, IL-17, and interferon (IFN)-γ in BAL fluid were determined by ELISA. OVA-specific IgE in BAL fluid was also detected by ELISA. Data are means ± standard deviations of three independent determinations with BAL cells from n = 5 mice/group.

Intracellular cytokine analysis of BAL fluid cells from OVA exposed mice revealed that TG2^−/−^ mice showed decreased amounts of IL-4- and/or IL-13-secreting CD4^+^ T cells compared to WT mice (Figure 3A). IL-4, IL-13, IL-17, and OVA-specific-IgE levels in the BAL fluid decreased, whereas interferon (IFN)-γ increased, in TG2^−/−^ mice compared to WT mice (Figure 3B).

### Cysteamine treatment reduced recruitment of airway inflammatory cells

**Figure 4:**
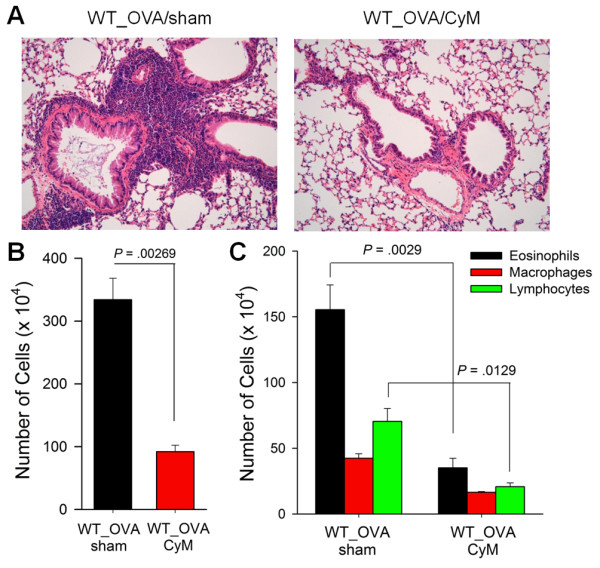
**Cysteamine treatment reduces airway hypersensitivity.** (A-C) WT mice were immunized with OVA plus alum and intranasally challenged with OVA. Mice were injected intraperitoneally with cysteamine (40 mg/kg/day) to inhibit TG2 activity. (**A**) Representative photographs of lungs from PBS- or cysteamine-treated mice. Sections are stained with hematoxylin and eosin (original magnification, ×200). (**B**, **C**) BAL fluid was obtained 48 h after the last challenge with OVA. BAL cells were counted and analyzed by Wright–Giemsa staining. Total (**B**) and differential cell counts (**C**) were performed. Data are means ± standard deviations of three independent determinations with BAL cells from n = 5 mice/group.

We intraperitoneally injected cysteamine twice daily (40 mg/kg) from the day of the first intraperitoneal OVA sensitization to evaluate the effect of the transglutaminase pharmacological inhibitor on the pathogenesis of allergic asthma. Cysteamine treatment decreased airway inflammation compared to that in the PBS-treated control (Figure 4A). The BAL fluid analysis revealed that cysteamine greatly reduced the number of infiltrating inflammatory cells compared to the PBS-treated control (Figure 4B). The decrease in inflammatory cells was not confined to eosinophils, as cysteamine treatment reduced all components of innate and adaptive cells recruited to the lung, including eosinophils, macrophages, and lymphocytes (Figure 4C).

### Cysteamine treatment reduced T helper cell differentiation

**Figure 5:**
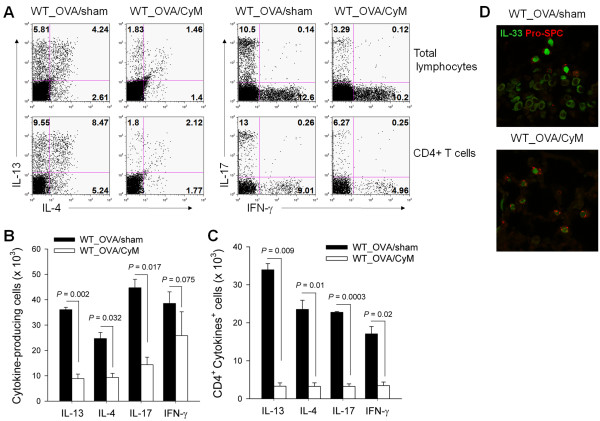
**Cysteamine treatment reduces T helper cell differentiation.** BAL cells were harvested 48 h after the last OVA challenge and restimulated with phorbol myristic acid (PMA) and ionomycin for 4 h. Intracellular cytokine levels were analyzed by flow cytometry. (**A**) The proportions of interleukin (IL)-4-, IL-13-, IL-17-, and interferon (IFN)-γ-producing cells are shown. (**B**) Number of cytokine-producing cells. (**C**) Number of cytokine-producing CD4^+^ T cells. (**D**) Immunofluorescence staining of IL-33 (green) and pro-surfactant protein C (SPC) (red) in the lungs from PBS or CyM-treated WT mice. Sections were prepared 24 h after the last ovalbumin (OVA) challenge (original magnification, ×1000). (**A**-**C**) Data are means ± standard deviations of three independent determinations with BAL cells from n = 5 mice/group.

Cysteamine (CyM) treatment reduced all cytokine-secreting lymphocytes tested when we analyzed BAL fluid infiltrating lymphocytes using intracellular cytokine staining. Not only IL-4-, IL-13-, and IL-17-secreting lymphocytes, but also IFN-γ-secreting lymphocytes decreased in percentage and number compared to those in the PBS-treated control (Figure 5A and 5B). T helper cell differentiation to all pathways tested also decreased following cysteamine treatment when we gated CD4^+^ T cells (Figure 5A and 5C). We assessed IL-33 expression from pulmonary epithelial cells following disease induction to evaluate the role of TG2 during the initiation of Th2 differentiation and airway hyperresponsiveness, as IL-33 has been implicated as the most upstream epithelial cytokine mediator leading to the Th2 phenotype [16]. PBS-treated WT mice revealed increased IL-33 expression in pulmonary epithelial cells following intranasal OVA challenge, whereas CyM-treated mice showed reduced IL-33 expression (Figure 5D).

### Reduced IL-33 expression in TG2-deficient mice

**Figure 6:**
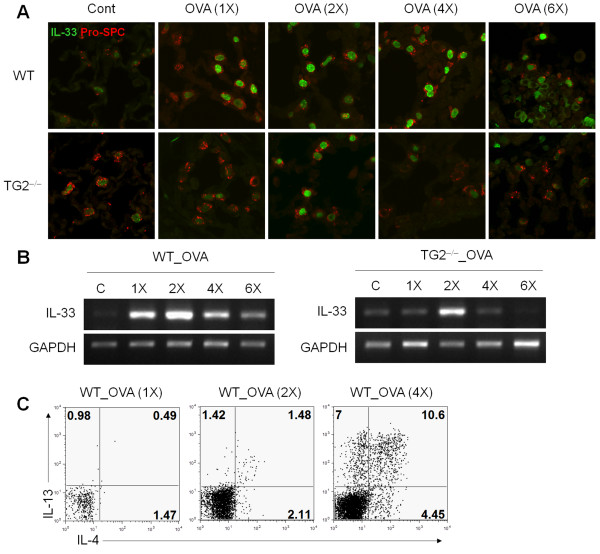
**Reduced IL-33 in TG2**^**−/− **^**mice.** (**A**) Immunofluorescence staining of IL-33 (green) and pro-SPC (red) in the lung from WT and TG2^−/−^ mice. Sections were prepared 24 h after OVA challenge. (original magnification, ×1000). (**B**) IL-33 mRNA expression in lung tissues of WT and TG2^−/−^ mice were determined by reverse transcription-polymerase chain reaction. (**C**) BAL cells were harvested 24 h after OVA challenge and restimulated with PMA and ionomycin for 4 h. Intracellular cytokine levels were analyzed by flow cytometry. The proportions of IL-4- and IL-13-producing cells are shown.

WT mice revealed increased IL-33 expression in pulmonary epithelial cells, whereas TG2^−/−^ mice showed reduced IL-33 expression (Figure 6A). The kinetics of IL-33 expression also showed delayed and reduced IL-33 expression in TG2^−/−^ mice compared to WT mice (Figure 6A). Reduced IL-33 expression in the TG2^−/−^ mice was also revealed by reverse transcription polymerase chain reaction (RT-PCR) analysis of the lung. TG2^−/−^ mice also showed reduced TSLP expression compared to WT mice (Figure 6B). Th2 responses occurred in the wild-type mice only following 4 times or more of intranasal OVA challenges, which indicated the Th2 responses developed when IL-33 expression reached its peak level (Figure 6C).

## Discussion

In this study, we observed that TG2 plays an important role in initiating the allergic Th2 response by inducing IL-33 and downstream molecules leading to Th2 differentiation following allergen sensitization and challenge. Airway hyperresponsiveness was attenuated in TG2-deficient mice compared to WT control mice, and recruitment of eosinophils and Th2 and Th17 differentiation decreased in TG2-deficient mice. We confirmed the role of TG2 in the pathogenesis of allergic asthma using cysteamine, a transglutaminase inhibitor. TG2-deficient mice revealed reduced IL-33 expression following asthma induction. Thus, we provide evidence that TG2 in pulmonary epithelial cells initiates allergic responses through the IL-33-Th2 signaling pathways.

The roles of TG2 in the pathogenesis of organ-specific and systemic inflammatory responses including hypersensitivity reactions have been documented. Cysteamine, a broad-spectrum transglutaminase inhibitor, reduces pulmonary inflammation and fibrosis following intratracheal bleomycin instillation [8]. Cystamine, a dimeric form of cysteamine, also ameliorates IgE-induced passive cutaneous anaphylaxis and phorbol myristic acid-induced atopic dermatitis [20]. Sohn *et al.*[21] developed recombinant peptides with dual inhibitory capacity against TG2 and phospholipase A2, and these recombinant peptides reversed the inflammation of allergic conjunctivitis. These authors also found that R2 peptide treatment ameliorates inflammatory allergic asthma induced by OVA sensi-tization and challenge [22]. A correlation between TG2 expression and disease progression has been suggested in patients with toluene diisocyanate-induced occupational asthma and exercise-induced bronchoconstriction [23,24]. Although an important role for TG2 in allergic asthma pathogenesis has been suggested by these reports, all studies used rather nonspecific pharmacological inhibitors or conducted relative expression studies. Therefore, the critical role of TG2 during asthma pathogenesis has not been properly evaluated using genetically modified mice. In this respect, our results utilizing TG2-deficient mice show a critical role for TG2 during the pathogenesis of experimental allergic asthma and suggest that TG2 is a putative novel disease target of allergic asthma.

Asthma is a chronic inflammatory disease with characteristics of type 2 cytokine production. IL-33 is a strong inducer of the Th2 immune response, and its role in the immunopathogenesis of allergic asthma had been documented. Higher IL-33 expression occurs in patients with asthma and in murine models of asthma [25,26]. Increased airway hyper-responsiveness and inflammatory cell infiltration is observed by either IL-33 administration or by studies with IL-33 overexpressing transgenic [27,28]. IL-33-deficient mice reveal reduced inflammatory cell recruitment to the lung and attenuated airway hyper-responsiveness compared to that of a WT control [29]. In addition, blocking ST2 signaling either by blocking antibodies or by using a soluble receptor protein inhibits pulmonary inflammation and airway hyperresponsiveness [30]. IL-33 induces type 2 cytokine production through the ST2 receptor expressed on multiple innate and adaptive immune cells, including type 2 innate lymphoid cells, Th2 cells, mast cells, basophils, eosinophils, and natural killer T cells [31]. Further, IL-33 stimulates the expression of TSLP and its receptor and, thus, indirectly induces Th2 responses [4,32], which was also reduced in TG2-deficient mice in our study. Data on the IL-33 induction signaling pathway are incomplete, but a recent report revealed a role for extracellular ATP in triggering IL-33 release following airborne allergen exposure [33]. In our study, expression of IL-33 increased in the type II pulmonary epithelial cells following intranasal OVA treatment and increased further following additional intranasal injection of OVA (Figure 6A). TG2 expression on the pulmonary epithelial cells also increased by intranasal OVA injection and further increased with additional OVA challenge, which closely paralleled IL-33 expression kinetics. Among the several suggested mechanisms of TG2, transglutaminase enzyme activity may be involved in the regulation of IL-33 expression in that inhibition of enzyme activity using CyM also decreased IL-33 expression comparable to TG2 knockout mice (Figure 5D). In addition, we found that Th2 responses occurred in the wild-type mice only following 4 times or more of intranasal OVA challenges in our experimental settings, which indicated the Th2 responses developed when IL-33 expression reached its peak level (Figure 6C). Thus we suggest that TG2 and downstream IL-33 expression seem to be important in the induction of Th2 response and eosinophil recruitment.

The important role of lung epithelial cells in initiating pulmonary inflammation during allergic responses requires further discussion. Epithelial cells are implicated as an important initiator and perpetuator of protective and pathological inflammation from both infectious and non-infectious stimuli [34,35]. IL-33 and its ST2 receptor are mainly expressed in bronchial epithelial cells in the lung [25]. Thus, airway epithelial cells are also active players in the pathogenesis of asthma through epithelial cytokines including IL-33, TSLP, and IL-25, which are produced and released by epithelial cells in response to various exogenous stimuli or by cellular damage [36]. In our previous report, epithelial TG2 played a critical role in initiating the inflammatory response leading to the type 17 response following bleomycin treatment [8].

## Conclusion

In this study, we found that pulmonary epithelial cells damaged by allergens triggered TG2-mediated IL-33 expression leading to type 2 responses by recruiting both innate and adaptive arms of the immune system. Thus, epithelial TG2 was a common critical link transducing epithelial tissue damage to initiate Th2 or Th17 inflammatory responses depending on the context of the stimuli.

## Abbreviations

TG2: Transglutaminase; BALF: Bronchoalveolar lavage fluid.

## Competing interests

The authors have declared that no conflict of interest exists.

## Authors’ contributions

KO designed the research, performed the experiments, interpreted the data and wrote the manuscript; MWS, GYL, and OJB performed the experiments; HRK and SHC interpreted the data and reviewed the manuscript; DSL designed the research, interpreted the data, wrote and edited the manuscript. All authors read and approved the final manuscript.
